# Urologic Fistulas in Czech Women With Gynaecologic Malignancies

**DOI:** 10.1002/cnr2.70134

**Published:** 2025-04-03

**Authors:** Jiri Spacek, Munachiso Onyedikachi Ndukwe, Akaninyene Eseme Bernard Ubom, I. Sirak, Petr Hoffman, Dominik Karasek, Jiri Petera, Milos Brodak, Michal Balik, Dominik Habes, Jaroslav Pacovsky

**Affiliations:** ^1^ Department of Urology University Hospital Hradec Kralove Hradec Králové Czech Republic; ^2^ Department of Obstetrics and Gynecology University Hospital Hradec Kralove Hradec Králové Czech Republic; ^3^ Department of Obstetrics, Gynaecology, and Perinatology Obafemi Awolowo University Teaching Hospitals Complex, Ile‐Ife Ile‐Ife Osun State Nigeria; ^4^ Department of Oncology and Radiotherapy University Hospital Hradec Kralove Hradec Králové Czech Republic; ^5^ Department of Radiology University Hospital Hradec Kralove Hradec Králové Czech Republic

**Keywords:** cancer, fistula, radiotherapy, surgery

## Abstract

**Introduction:**

In developed countries, urologic fistulas arise mainly from malignancies, radiotherapy, or surgical trauma. Hysterectomy and radiation therapy are both critical components of the treatment of women with cancers. Urologic fistulas significantly reduce the quality of life of cancer patients, and may result in delays or even refusal of adjuvant treatment by these patients, thereby negatively impacting both short‐ and long‐term cancer survival.

**Materials and Methods:**

A 10‐year retrospective study of urologic fistulas associated with gynaecologic malignancies at the University hospital Hradec Kralove, Czech Republic was conducted. Descriptive statistics of the fistula and treatment characteristics of women with malignant fistulas were conducted using the NCSS 22 statistical software program (NCSS, Keysville, Utah).

**Results:**

Cervical cancer was mostly commonly associated with urologic fistulas (36, 76.8%). Most of the malignant fistulas were complex (41, 87.2%) vesicovaginal (23, 48.9%) fistulas (VVFs). More than two‐thirds (33, 70.2%) of the fistulas were diagnosed following radiotherapy, with a time interval from radiotherapy to fistula diagnosis of between 3.00 and 14.50 years. Primary fistuloraphy was performed for all the six cases with simple VVFs and seven (41.2%) of the 17 patients with complex VVFs. Treatment success rate was 83.33% and 14.3% for simple and complex fistulas, respectively. All the failed complex fistula repairs recurred.

**Conclusion:**

Malignant fistulas predominantly follow radiotherapy for cervical cancers, and are usually detected up to 15 years post‐radiotherapy. Most are complex VVFs, which are difficult to treat, with a high rate of recurrence.

## 1 Introduction

Urologic fistula is a pathological communication between the urinary tract and surrounding organs [1]. In developing countries, urologic fistulas are mainly of obstetric origin, mostly associated with prolonged or obstructed labour, whereas in developed countries, they are usually related to genitourinary (GU) injuries following pelvic surgery [2]. GU injury is estimated to occur at a rate of 1%–2% for all major gynecologic surgeries, with 75% of these injuries occurring during hysterectomy [3]. The incidence of ureteral injury following hysterectomy is 0.03%–1.5% and for bladder injuries, 0.2%–1.8% [1]. More than 70% of these injuries are not recognised at the time of surgery. Mechanisms of GU fistula formation following gynecologic surgery include unrecognised iatrogenic cystotomy during dissection, poor repair of cystostomy or breakdown of cystostomy repair, iatrogenic incorporation of the bladder with surrounding structures during suturing, devascularization and thermal injuries [4]. About 8% of GU fistulas of surgical aetiology follow radical hysterectomy [5]. Overall, the incidence of urologic fistula following hysterectomy is 0.1%–4% [4]. Radiotherapy is another risk factor for urologic fistulas. In the United States of America, about 100 000 patients received radiation therapy for gynaecological malignancies in 2014, with at least 4% of them developing radiation‐induced GU fistulas [6].

Genitourinary fistulas significantly reduce the quality of life of affected patients, lead to higher secondary surgery rates, higher readmission rates and increased healthcare costs. They may also result in delays or even refusal of adjuvant treatment by patients with malignancies, thereby negatively impacting both short‐ and long‐term cancer survival [2, 5]. Hysterectomy and radiation therapy are both critical components of the treatment of women with gynecologic malignancies. Given the risks of urologic fistulas associated with both, and the significant negative impacts of urologic fistulas on the treatment and survival of women with gynaecologic malignancies, we undertook a retrospective review to determine the prevalence and associated morbidity of urologic fistulas in women with gynaecologic malignancies in a tertiary urologic and cancer facility in Czech Republic.

## 2 Materials and Methods

**Study design, setting and period:** A retrospective study of urologic fistulas associated with gynaecologic malignancies at the University hospital Hradec Kralove, Czech Republic, was conducted over a 10‐year period, from 1 January 2011 to 31 December 2020.

**Study inclusion and exclusion criteria:** All cases of urologic fistulas seen in adult women aged ≥ 18 years, who had a history of a gynecologic malignancy, or who had been recently diagnosed or being treated for a gynecologic cancer at the time of fistula diagnosis; were included the study. All types of gynaecologic malignancies were included. All cases with missing or incomplete records were excluded from the study.

**Data collection:** The case records of all patients who presented with urologic fistulas during the study period were retrieved, and relevant data extracted from the case records using a purpose‐designed proforma. Relevant information recorded included types of fistulas and associated malignant and benign etiological conditions, cancer treatment prior to fistula diagnosis, time interval from cancer treatment to fistula diagnosis, fistula treatment modalities and their success rates, associated upper urinary tract complications and treatment. The urologic fistulas were classified both based on anatomy and the degree of operative difficulty into simple with good prognosis and complex fistulas with uncertain prognosis as per the World Health Organisation (WHO) 2006 classification system [7].

**Statistical analysis:** Descriptive statistics of the fistula and treatment characteristics of women with malignant fistulas were conducted using the NCSS 22 statistical software program (NCSS, Keysville, Utah). Categorical variables were presented as frequencies and percentages while continuous variables were presented as mean and standard deviation.

**Ethics:** Ethical approval for this study was obtained from Institutional Research Board (IRB) of University hospital Hradec Kralove, Czech Republic.

## 3 Results

### 3.1 Types of Urologic Fistulas and Associated Malignant and Benign Conditions

During the study period, a total of 64 urologic fistulas were recorded, with 47 (73.4%) of these associated with gynecologic malignancies. Most (39, 60.9%) of the fistulas occurred in women aged 50 years and above. The mean age of the women with malignant fistulas ranged from 57.2 ± 15.6 years for women with cervical cancer to 63.3 ± 15.7 years for women with ovarian cancer. The most common gynecologic cancer associated with urologic fistula was cervical cancer (36/47, 76.6%), while the least common was vaginal cancer, seen in only one case. The predominant type of malignant fistula was vesicovaginal (23, 48.9%). An overwhelming majority (41, 87.2%) of the malignant fistulas were complex fistulas (Table 1). All six simple fistulas seen within the study period were vesicovaginal. Twenty‐seven (57.4%) of the 47 malignant fistulas were diagnosed with the patients in remission, while the remaining 20 (42.6%) cases presented either during recurrence or the primary tumour manifestation.

**TABLE 1:** Types of urologic fistulas and associated malignant and benign conditions.

Characteristic	Frequency, *n =* 64	Percent (%)
Diagnosis		
*Malignancies* (*n* = 47)		
Cervical cancer	36	56.3
Ovarian cancer	6	9.4
Endometrial cancer	4	6.3
Vaginal cancer	1	1.6
*Benign conditions* (*n* = 17)		
Uterine leiomyoma	11	17.2
Injuries of abdomen, lower back, pelvis and external genitals	2	3.1
Caesarean section	2	3.1
Urethral diverticulum	1	1.6
Endometriosis	1	1.6
Type of malignant fistula (*n =* 47)		
Vesicovaginal	23	48.9
Vesicointestinal	8	17.0
Vesicorectovaginal	8	17.0
Ureterovaginal	3	6.4
Uretero‐iliac/uretero‐aortic	3	6.4
Vesicocutaneous	2	4.3
WHO classification of malignant fistula (*n = 47*)		
Simple	6	12.8
Complex	41	87.2

### 3.2 Types of Malignant Urologic Fistulas, Prior Cancer Treatment and Time Interval From Cancer Treatment to Fistula Diagnosis

Thirty‐three (70.2%) of the malignant fistulas were diagnosed following radiation therapy, while 23 (47.9%) followed radical hysterectomy. Majority (21, 44.7%) of the malignant fistulas followed radiotherapy or chemoradiotherapy alone, while 11 (23.4%) cases were diagnosed following surgery alone and 12 (25.5%) cases followed a combination of surgery and radiotherapy (Table 2). One‐half (18/36, 50%) of the women with cervical cancer developed malignant fistula following primary radiotherapy. The time interval from cancer treatment to fistula diagnosis was shortest when treatment involved surgery alone, between 0.03 and 0.67 years, compared to between 3.00 and 14.50 years when treatment involved radiotherapy (Table 2).

**TABLE 2:** Types of malignant urologic fistulas, prior cancer treatment and time interval from cancer treatment to fistula diagnosis.

Fistula type	*n*	Cancer treatment prior to fistula diagnosis	Time from end of cancer treatment to fistula diagnosis (years)
Vesicovaginal	15 3 5	46.7% (*n* = 7/15)—Radical hysterectomy and adjuvant radiotherapy 40.0% (*n* = 6/15)—Radiotherapy or chemoradiotherapy 13.3% (*n* = 2/15)—No previous treatment^a^ Radical surgery Radical debulking surgery^b^	14.50 0.24 0.67
Ureterovaginal	3	Radical hysterectomy	0.03
Uretero‐iliac/Uretero‐aortic	3	66.7% (*n* = 2/3)—Chemoradiotherapy 33.3% (*n* = 1/3)—Radical hysterectomy and adjuvant radiotherapy	11.67
Vesicointestinal	1 6 1	Radiotherapy and brachytherapy Radiotherapy or chemoradiotherapy Chemotherapy^c^	5.00 9.91 2.00
Vesicorectovaginal	7 1	85.7% (*n* = 6/7)—Radiotherapy or chemoradiotherapy 14.3% (*n* = 1/7)—Radical hysterectomy and adjuvant radiotherapy Radical hysterectomy and adjuvant radiotherapy	13.60 3.00
Vesicocutaneous	2	Radical hysterectomy and adjuvant radiotherapy	7.50

^a^ Primary locally advanced tumour with vesicovaginal fistula; tumour and fistula diagnosis at the same time.

^b^ Hysterectomy with adnexectomy, lymphadenectomy, omentectomy; 80% of fistulas manifested within 1 month after surgery.

^c^ The vesicointestinal fistula manifestation at the time of tumour recurrence.

### 3.3 Treatment Modalities According to Fistula Type, Treatment Success Rates and Complications

All six patients with simple vesicovaginal fistulas (VVFs) underwent primary fistuloraphy, with a success rate of 83.33% (5/6). Conversely, seven (41.2%) of the 17 patients with complex VVFs underwent primary fistuloraphy, with only one (14.3%) being successful; a fistula recurrence occurred in the rest of the patients (Table 3). Of the two uretero‐iliac fistulas, treatment was successful in one, while the other was fatal. Treatment for the uretero‐iliac fistulas included vasography with embolization and stent graft insertion. Aortic graft surgery was done for the single case of uretero‐aortic fistula, which was complicated with fatal bleeding and exsanguination. Fistuloraphy was performed in eight cases within 1 month from the previous cancer surgery. In the remaining cases, surgical repair was delayed until a time gap of at least 5 months. Thirty‐four patients (72.3%) with malignant fistulas presented with unilateral or bilateral hydronephrosis, with 27 (79.4%) of these requiring urinary diversion in the form of ureteral stent or nephrostomy (Table 4).

**TABLE 3:** Treatment modalities according to malignant fistula type and treatment success rates.

Type of fistula	Primary treatment modality of fistula	Fistula treatment success rate
Vesicovaginal Simple (26%) Complex (74%)	Transvaginal, transperitoneal (open, laparoscopic or robotic assisted) fistuloraphy Transvaginal, transperitoneal (open) fistuloraphy; Urinary diversion (ureteroileostomy)	83.33% 14.29% 100%
Ureterovaginal	Ureterocystoneostomy (open)	100%
Uretero‐iliac	Vasography with embolization and stent graft insertion	50%
Uretero‐aortal	Aortic graft surgery	^a^
Vesicointestinal or Vesicorectovaginal	In 33.3% of cases‐terminal stomy (trasversostomy, colostomy or sigmoideostomy) In 66.7% of cases‐no treatment due to poor health status	^b^
Vesicocutaneous	Urinary bladder augmentation	^c^

^a^ Fatal bleeding with exsanguination during reoperation.

^b^ 81.3% of patients‐ urinary diversion by unilateral or bilateral nephrostomy.

^c^ Reoperation with ureteroileostomy as definite solution in both cases.

**TABLE 4:** Upper urinary tract complications and types of urinary diversion associated with malignant fistulas.

Upper urinary tract complication	Frequency, *n =* 34	Type of urinary diversion
Unilateral hydronephrosis	14	Stent (*n =* 4) Nephrostomy (*n =* 6) Without diversion (*n =* 4)
Bilateral hydronephrosis	20	Unilateral diversion (stent or nephrostomy) (*n =* 7) Bilateral diversion (stent or nephrostomy) (*n =* 10) Unknown (*n =* 3)

## 4 Discussion

The most common gynecologic cancer associated with urologic fistula in this study was cervical cancer. Radical surgery (including radical abdominal hysterectomy, laparoscopic assisted vaginal or total laparoscopic radical hysterectomy and radical abdominal trachelectomy) is recommended for early‐stage cervical cancer (FIGO stages 1B and 2A) [8]. Surgery produces good survival outcomes and obviates the morbidity of chemo‐radiation. However, extensive dissection of the ureters and bladder during these radical surgeries heightens the risk of intra‐operative GU injuries and post‐operative fistulas. The incidence of urinary fistulas following radical surgery for cervical cancer varies from 0.6% to 5.1% [9]. The mean ages of occurrence of urologic fistula in women with cervical and ovarian cancers in our study corresponds to the reported peak ages of incidence of both cancers‐ sixth decade of life for cervical cancer and seventh to eighth decade of life for ovarian cancer [10, 11]. As similarly reported in the literature [12], VVFs were the predominant fistulas in our study. Whereas VVFs are commonly due to prolonged/obstructed labours in developing countries, in developed countries like ours, they arise mainly from malignancies, radiotherapy, or surgical trauma [13]. When cervical cancer (the most common cancer in our study) involves bladder invasion (stage IVA), development of VVF is frequent, occurring in up to 50% of cases [14]. Conversely, uretero‐iliac/uretero‐aortic fistulas are rare and often life threatening, with a reported associated mortality rate of 7%–23% [15]. They constituted less than one‐tenth of the malignant fistulas in our study, with a mortality of 67%.

Radiotherapy, chemotherapy and surgery are the three main treatment modalities for gynaecologic malignancies. The recommended treatment for locally advanced cervical cancer, which was associated with more than three‐fourth of the malignant fistulas in this study, is chemoradiation [14]. Owing to locoregional anatomy, the bladder, urethra, and distal ureters usually receive some of the radiation dose during pelvic radiotherapy, resulting in acute and late side effects, including radiation cystitis, urinary strictures and fistula formation. Radiation‐induced fistulas are usually vesico‐vaginal, as also seen in this study, with an incidence rate of 1%–10% following pelvic radiotherapy [16]. This rate increases to 14.5%–17% following radiotherapy for cervical and endometrial cancers [16], both cancers were responsible for more than four‐fifth of the malignant fistulas in this study. One‐half of the women with cervical cancer in this study developed fistulas following primary radiotherapy treatment. Radiation causes endarteritis, resulting in vascular occlusion, ischemia, necrosis and fibrosis. Fibrosis of the ureteric or bladder mucosa leads to loss of compliance, and consequently an increase in wall tension. This, together with tissue necrosis, leads to fistula formation [16, 17]. Radiation‐induced fistulas are usually large (> 10 mm), multiple (≥ 2), and complex, with low likelihood of spontaneous closure [17, 18]. This may explain why more than four‐fifth of the fistulas in this study were complex fistulas.

For post‐operative urologic fistulas, the diagnosis is usually made within a few days to weeks after surgery, while post‐radiation urologic fistulas can occur any time after 6 months to 20 years after radiation therapy [18]. This was evident in this study, where postsurgical fistulas occurred within a year of surgery, compared to post‐radiation fistulas that were diagnosed between three to 15 years post‐radiotherapy. An ideal timing of fistula repair is very important, as the first repair attempt is the best attempt and has the highest probability of achieving successful outcome [19]. Typically, it is recommended to delay surgical repair for 12 weeks to allow inflammation to subside and for the necrotic tissues to slough out [13, 19]. Early repair, however, reduces psychological distress on the women, and can be performed in the absence of infection. Contraindications to early repair include radiation‐induced fistula and associated enteric injury [13]. The impaired vascularity and scarring associated with radiation exposure make attempts at repair of radiation‐induced fistulas more challenging [20]. Radiation‐induced reaction takes about 1 year to heal; it is, therefore, recommended that repair of radiation‐induced fistulas should be delayed for 6–12 months [19, 20]. In this study, surgical repair was delayed in the majority of cases until a time gap of at least 5 months. A treatment success rate of 25% has been reported for radiation‐induced fistulas [21]. In this study, whereas treatment was successful in more than 80% of simple fistulas, less than 15% of complex fistulas (which constituted more than 85% of all the fistulas) were successfully treated, with a recurrence in the rest of the cases.

## 5 Conclusion

Malignant fistulas in women with gynaecologic malignancies are mostly seen in the sixth and seventh decades of life. They predominantly follow radiotherapy for cervical cancers, and are usually detected up to 15 years post‐radiotherapy. Most are complex VVFs, which are difficult to treat, with a high rate of recurrence. To optimize outcomes, treatment of radiation‐induced urologic fistulas in women with gynaecologic cancers should be delayed for 6–12 months, to allow adequate time for radiation‐induced reaction to heal before repair.

## 6 Author Contributions

Conceptualization: Jiri Spacek and Jaroslav Pacovsky. Methodology: Jiri Spacek, Munachiso Onyedikachi Ndukwe, Akaninyene, Eseme Bernard Ubom, Igor Sirak and Jaroslav Pacovsky. Formal analysis: Jiri Spacek, Akaninyene Eseme Bernard Ubom and Jaroslav Pacovsky. Data curation: Jiri Spacek, Petr Hoffmann, Dominik Karasek, Michal Balik, Dominik Habes and Jaroslav Pascovsky. Investigation: Petr Hoffmann, Dominik Karasek, Michal Balik, Dominik Habes. Writing – original draft: Jiri Spacek, Jaroslav Pacovsky and Igor Sirak. Writing – review and editing: Jiri Spacek, Munachiso Onyedikachi Ndukwe, Akaninyene Eseme Bernard Ubom, Jiri Petera and Milos Brodak. Software: Igor Sirak. Funding acquisition: Jiri Petera and Milos Brodak. Supervision: Jaroslav Pacovsky. All authors had full access to the data in the study and take responsibility for the integrity of the data and accuracy of the analysis.

## 7 Conflicts of Interest

The authors declare no conflicts of interest.

## Data Availability

The data that supports the findings of this study are available in the Supporting Information of this article.
